# A Comprehensive Updated Review of Pharmaceutical and Nonpharmaceutical Treatment for NAFLD

**DOI:** 10.1155/2016/7109270

**Published:** 2016-02-23

**Authors:** Newaz Hossain, Pushpjeet Kanwar, Smruti R. Mohanty

**Affiliations:** Department of Gastroenterology, New York Methodist Hospital, Brooklyn, NY 11215, USA

## Abstract

Nonalcoholic fatty liver disease (NAFLD) is the most common liver disease in the western world with prevalence of 20–33%. NAFLD comprises a pathological spectrum. Nonalcoholic fatty liver (NAFL) is at one end and consists of simple hepatic steatosis. On the contrary, nonalcoholic steatohepatitis (NASH) consists of steatosis, inflammation, and ballooning degeneration and can progress to cirrhosis. Despite the rising incidence, definitive treatment for NAFLD, specifically NASH, has not yet been established. Lifestyle modification with dietary changes combined with regular aerobic exercise, along with multidisciplinary approach including cognitive behavior therapy, has been shown to be an effective therapeutic option, even without a significant weight loss. Pioglitazone and vitamin E have shown to be most effective in NASH patients. Surgery and weight loss medication are effective means of weight loss but can potentially worsen NASH related fibrosis. Other agents such as n-3 polyunsaturated fatty acids, probiotics, and pentoxifylline along with herbal agent such as milk thistle as well as daily intake of coffee have shown potential benefits, but further well organized studies are definitely warranted. This review focuses on the available evidence on pharmaceutical and nonpharmaceutical therapy in the treatment and the prevention of NAFLD, primarily NASH.

## 1. Introduction

Nonalcoholic fatty liver disease (NAFLD) is a spectrum of liver disease characterized by macrovesicular fatty infiltration, inflammation, and hepatocytes ballooning injury. It has become the most common cause of liver disease and abnormal liver function test in industrialized countries [1, 2] with a prevalence of 20 to 33% [3, 4]. There is lower prevalence and lower degree of liver cell injury in African American population while higher prevalence is reported in Asians and Hispanics with advanced grade of liver damage compared to other ethnicities [5]. Approximately 30% of patients with simple steatosis or nonalcoholic fatty liver (NAFL) will eventually develop nonalcoholic steatohepatitis (NASH) [6, 7], while 15–25% of NASH patients will eventually progress to cirrhosis and 30–40% of cirrhotic patients will die due to liver related complications [8]. Most common cause of death in patients with NAFLD is cardiovascular disease, followed by extra hepatic cancer, with the third being liver disease [9–11].

Pathophysiology of NAFLD is still unclear; however, knowledge about pathophysiology would help understand potential therapies. Pathogenesis is believed to be largely due to liver insults starting with development of insulin resistance in patients with central adiposity or metabolic syndrome risk factors. This insulin resistance leads to increased serum free fatty acids (FFA) that causes lipogenesis in the liver and thus leads to hepatic steatosis [12]. Transformation from simple steatosis to steatohepatitis is unclear, but many complex mechanisms have been hypothesized which work in conjunction with hepatic necroinflammation and ballooning in steatotic livers. Circulating FFA activate number of intrinsic and extrinsic pathways leading to activation of proapoptotic proteins that lead to oxidative stress and apoptosis, eventually causing NASH.

Patients with NAFLD often remain asymptomatic and therefore most of them are undiagnosed for many years until eventually progressing to advanced fibrosis or cirrhosis and hepatocellular carcinoma. However, patients may sometimes present with vague right upper quadrant pain, hepatomegaly, and normal or high alanine transaminase (ALT) and usually have risk factors for metabolic syndrome. Up to 70–80% of individuals with NAFLD have insulin resistance or metabolic syndrome [13]. Although NASH is associated with risk factors of metabolic syndrome such as diabetes and obesity, thin patients with normal waist circumferences can also have NASH but they usually present with higher ALT levels [14]. There is no optimal ALT level to predict the extent of NASH and advanced fibrosis [15], but NAFLD is the most common cause of incidental elevation in liver enzymes [16]. However, laboratory testing and radiological imaging cannot differentiate NASH from NAFL. Abdominal ultrasound is most commonly used to detect liver echogenicity, but it is highly operator dependent and lacks diagnostic sensitivity [17]. Currently, gold standard for diagnosis of NASH is liver biopsy, but it is invasive in addition to having high probability of sampling error [18]. Although noninvasive methods such as NAFLD fibrosis score, FIB-4, NAFIC score, and HAIR score as well as imaging modalities such as transient elastography are useful diagnostic methods for evaluating steatohepatitis or fibrosis [19], liver biopsy is still the definitive diagnostic method for confirming a diagnosis of NASH.

Despite growing understanding of the global epidemic of NAFLD, there is no definite pharmacotherapy available. Only proposed beneficial treatment according to available evidence is lifestyle modification with regular exercise and dietary changes or bariatric surgery in morbidly obese patients leading to gradual weight loss [20–22]. Other suggested means of pharmacological therapy are anti-inflammatory or antioxidant agents. Although insulin sensitizers such as thiazolidinediones and antioxidants such as vitamin E have shown promising results, there is some concern for adverse effects and long-term safety. However, these agents should be used in addition to lifestyle modification. Most available data on pharmacologic interventions consists of small nonrandomized studies which lack a control group and histological evaluation. This review article will highlight the current evidence on pharmacological and nonpharmacological therapeutic options for NAFLD.

## 2. Lifestyle Intervention

Obesity and metabolic syndrome are the most important risk factors for NAFLD [23]. Excessive food intake, particularly western diet that includes complex carbohydrates and saturated fats along with sedentary lifestyle, has increased the prevalence of obesity and metabolic syndrome leading to NAFLD. Therefore, lifestyle intervention targeting weight loss has been of great interest in terms of being a therapeutic option for NAFLD (see Table 1). Nevertheless, adjusting such changes to one's lifestyle and achieving the target weight reduction are difficult and many factors may play a role. Furthermore, weight loss of at least 7% is required for histological improvement [24] and greatest improvement achieved with >10% weight loss [25]; however, only 50% of patients are able to achieve that goal [26, 27]. In addition, psychosocial factors may play a major role in achieving weight loss. Hence, multidisciplinary approach addressing psychosocial needs and behavioral support may be required to achieve sustained lifestyle changes for patients with NAFLD. In a study, Stewart et al. evaluated the usefulness of multidisciplinary approach by investigating the behavior change in patients with NAFLD and the impact of psychosocial factors and concluded that although readiness for behavior change did not predict subsequent change in weight, personality factors and psychiatric symptoms such as depression, low consciousness, and neuroticism were associated with decreased weight reduction [28]. Furthermore, nutritional counseling and advice on physical activity alone have been shown to significantly improve liver enzymes and metabolic risk factors [29], as well as histological parameters such as steatosis in biopsy proven NASH patients [30].

Even though weight reduction is the most important treatment strategy for NAFLD, rapid weight loss can even worsen liver disease [31, 32]. Multiple studies have demonstrated that weight loss leads to normalization of metabolic disturbances and improvement in liver chemistries, steatosis, necroinflammatory changes and fibrosis [30, 33, 34]. Although weight loss is the mainstay of treatment, healthy diet and physical activity alone may have benefits even without significant weight reduction [35–39].

### 2.1. Dietary Modification

It is important to recognize the role of nutritional imbalance between saturated and unsaturated fatty acids, as well as carbohydrates and proteins, in development and progression of NAFLD. Studies show that dietary deficiency of polyunsaturated fatty acids (PUFA) can lead to NAFLD [40] and patients with NASH seem to have overall decreased intake of PUFA and increased intake of saturated fat [41] and transfatty acids [42]. Furthermore, fructose is another common ingredient used as sweetener in many food products and soft drinks, which is found to have a strong association with development of metabolic risk factors and 2-3-fold increased risk of developing liver steatosis [43–45]. In a recent questionnaire study involving 427 patients with NAFLD, increased consumption of fructose was significantly associated with higher stage of hepatic fibrosis while in the older aged group (>48 years old) it was also associated with higher rate of hepatic inflammation and ballooning [46]. On the contrary, oligofructose (subgroup of inulin, which is not digested in the upper GI tract) consumption has the potential to cause weight loss and decrease inflammation of hepatocytes, as seen in a randomized double-blind, placebo-controlled trial, involving 48 healthy adults who received 21 grams of oligofructose daily versus placebo for 12 weeks [47]. On the other end of dietary habit spectrum, protein deficiency and malnutrition have also been shown to cause NASH [48]. Another culprit used as an additive to enhance flavor, monosodium glutamate, has also been shown to be associated with increased risk of fatty liver and inflammation within the hepatocytes [49]. To summarize, saturated fatty acids should be limited to 7–10% of total energy intake [42], while there should be increased consumption of food rich in mono- and polyunsaturated fatty acids. The carbohydrate intake should be limited to 40–50%, fat intake less than 30%, and protein intake 15–20% of total energy intake [50]. Additionally, fructose rich diet and additives such as monosodium glutamate should be avoided.

Dietary modification such as caloric restriction as well as carbohydrate and fat restrictions may help with improvement in liver enzymes, metabolic risk factors and hepatic steatosis. There is no consensus on the exact amount of calorie restriction that is required to achieve enough weight loss to have an effective response on NAFLD. Weight loss achieved through caloric restriction leads to reduced liver size and fat content [51] and may be able to reverse hepatic steatosis [52] and lead to overall histological improvement [30]. Studies on the effect of hypocaloric diet are small and nonrandomized and lack histological analysis and thus we cannot be assured regarding the benefits from such an approach [35, 53]. Few studies have evaluated the role of weight loss through dietary modification with focus on restricting carbohydrate and fat component. In one study, 170 overweight or obese subjects were randomized to either reduced carbohydrate or reduced fat diet for 6 months along with total calorie restricted diet (30% less energy intake/day) for all participants [54]. Both groups had decreased body weight, total body fat, visceral fat, and intrahepatic lipid content with no significant difference between the two groups. Similarly, a study of 59 women with NAFLD, randomized to low fat versus low carbohydrate diet for 6 months, showed that both groups had weight loss (5.5% and 5.7%, resp.) and both led to decreased liver enzymes [55]. There were no significant differences between the groups. Therefore, these studies suggest that hypocaloric diet, when combined with reduced carbohydrate or fat component, has a greater benefit in improving intrahepatic lipid content and liver enzymes.

Different dietary options with balanced macronutrient composition have been studied because of their effect on insulin resistance, weight loss, and lipid profile. Mediterranean diet consisting of nuts, fruits, vegetables, olive oil, fish, legumes, and so forth has been shown to reduce risk of metabolic syndrome, major cardiovascular events, and total mortality [56–58]. In one study including 73 patients with a diagnosis of NAFLD and 58 healthy controls, the relationship of NAFLD severity with adherence to Mediterranean diet was evaluated [59]. There was a significant negative correlation between adherence to Mediterranean diet and ALT, insulin levels, stage of fibrosis, severity of steatosis, and the likelihood of having NASH. Similarly, a randomized study comparing Mediterranean-like diet with diet consisting of 50–60% carbohydrate, 15–20% protein, and total fat <30% for two years showed significantly lower insulin resistance in the Mediterranean-style diet group [60]. Therefore, Mediterranean diet has shown promising results in controlling metabolic risk factors and possibly helping reverse steatosis in NAFLD patients, though further larger randomized controlled trials are needed to verify these results.

### 2.2. Exercise Alone

Physical activity, especially aerobic exercise, has been shown to improve steatosis and the intensity is directly related to the improvement in metabolic risk factors. In one study, 23 obese adults with sedentary lifestyle were randomly assigned to 4 weeks of aerobic exercise (cycling) versus regular stretching (placebo) [38]. In the aerobic exercise group, there was a significant reduction in mean hepatic triglyceride concentration, visceral adipose tissue, and plasma free fatty acids. Similarly, another study showed the same beneficial effect with aerobic exercise over a longer duration (12 weeks) [61]. Interestingly, both of these studies showed that aerobic exercise training reduces hepatic and visceral lipids even without weight loss. Additionally, aerobic exercise is shown to be far superior to resistance training, when it comes to lowering visceral adipose tissue (VAT) [62]. However, recent studies have shown that both the intensity and regularity of exercise are critical in reversing the histology in NASH patients. In one retrospective analysis of 813 adults with biopsy proven NAFLD (data collected from Nonalcoholic Steatohepatitis Clinical Research Network), subjects were categorized into inactive, moderate, or vigorous exercise based on self-reported time spent in physical activity [63]. Histological analysis showed that neither moderate intensity nor total exercise per week was associated with lower odds of having NASH or fibrosis. Instead, vigorous exercise was associated with decreased odds of having NASH and exceeding the US guidelines of vigorous exercise was associated with decreased odds of liver fibrosis.

### 2.3. Dietary Modification Plus Physical Activity

Dietary modifications such as calorie restriction as well as carbohydrate and fat restriction in combination with exercise have been assessed in multiple studies and combination strategy has shown better results. In a prospective study involving 261 patients with biopsy proven NASH, lifestyle modification with hypocaloric diet and moderate intensity exercise show that weight loss of >5% was associated with reduction of NAS and 90% of the participants with >10% weight loss had resolution of NASH [25]. Additionally, weight loss correlated with improvement in liver enzymes and other metabolic risk factors. In one randomized controlled trial, 56 participants were randomly assigned to one of four groups, standard care (physician advice alone), low fat diet plus moderate exercise, moderate fat/low carbohydrate diet plus moderate exercise, or moderate exercise alone for 6 months [64]. All participants had liver biopsy before and after study. Out of the 41 patients who completed the study, 36 had NASH at baseline. All groups showed significant decrease in NAS and improvement in aminotransferases with no significant difference among the subgroups. Interestingly, majority of the population did not experience significant weight loss and in fact several participants actually gained weight and still had histological improvement. In another randomized control trial involving 31 biopsy proven NASH subjects assigned to either intense lifestyle modification including diet plus 200 minutes per week of moderate physical activity or dietary modification alone for 48 weeks [24]. Results showed that exercise and diet group had average of 9.3% weight loss versus 0.2% in the counseling group. Greater percentage (67%) of participants in the exercise group had reduction of NAS of ≥ 3 points or a posttreatment NAS of ≤2 compared to control group. There were no significant changes in the degree of hepatic fibrosis. However, this study did not have any participants with baseline advanced liver fibrosis. Nevertheless, this study showed that at least 7% weight loss is required to see a beneficial effect on liver histology. Similarly, in another study 96 overweight or obese adults with type 2 diabetes were randomly assigned to an intensive lifestyle intervention (calorie restriction and 175 minutes per week of moderate intensity physical activity) versus a control group who received education alone for 12 months [65]. Out of the 96 participants, 44% were found to have hepatic steatosis at baseline on magnetic resonance spectroscopy (MRS). Intense intervention group had significant reduction in BMI, waist circumference, weight (8.3%), total fat, and intraperitoneal fat. Compared with control group, the intensive group had significant decrease in steatosis as measured by MRS and those with >10% weight loss had even higher reduction in steatosis. Similarly, in a large study of 1,006 NAFLD patients, participants were randomly assigned to lifestyle intervention (diet and exercise) or control group for 12 months. There was significant improvement in waist circumference, BMI, ALT, metabolic markers, and homeostasis model assessment of insulin resistance (HOMA-IR) in the intervention group [86]. In summary, combination of diet and exercise has greater benefit than diet or exercise alone. Approximately 5% to 10% weight loss with diet and exercise has been shown to have beneficial effect on insulin resistance, serum transaminases, and liver histology.

## 3. Bariatric Surgery

Weight loss through bariatric surgery has been proposed as a possible treatment option for nonalcoholic fatty liver disease because of its positive effect on liver histology. Nevertheless, rapid sudden weight loss such as a result of bariatric surgery may lead to progression of liver failure in some NAFLD patients [31]. Numerous studies have shown improvement of steatosis and inflammation in NAFLD patients after bariatric surgery. There is still limited data on the effectiveness of bariatric surgery in NAFLD patients mainly due to lack of randomized controlled trials. Nevertheless, there are several retrospective and prospective cohort studies that have compared liver histology and metabolic markers before and after surgery.

A French study looked at the metabolic markers and histology before bariatric surgery and 1 year and 5 years after the surgery in 381 patients with severe obesity [67]. This study showed an improvement in steatosis, ballooning, and overall NAS with significant reduction in percentage of NASH patients at 5 years compared to before surgery (27.4% to 14.2%). The resolution of NASH at 1 year was significant (*p* < 0.00001), while there was no significant difference between 1 and 5 years. Interestingly, five years after surgery there was significant worsening of fibrosis in 19.8% of patients, but 95.7% of those with worsening fibrosis had fibrosis score ≤ F1. It is unclear as to the reason for worsening fibrosis, but point to be considered is that patients with worsening fibrosis were also those with higher BMI, steatosis, ballooning, inflammation, and more insulin resistance. Furthermore, BMI loss was not associated with fibrosis resolution. In another retrospective study, 78 morbidly obese patients underwent gastric bypass with liver biopsy performed during the surgery and after weight loss [66]. All patients had steatosis and 44.8% of them had evidence of fibrosis at baseline. Biopsy after weight loss reduction showed lower prevalence (30.8%) of hepatic fibrosis and a decrease in number of patients with NASH. In a meta-analysis, authors looked at 766 paired liver biopsies from 15 different studies [22]. The combined results showed that 91.6% of patients had improvement in steatosis, 81.3% of patients had improvement in steatohepatitis, and 65.5% of them had improvement in fibrosis, while 69.5% showed complete resolution of NASH. Although, in this meta-analysis, bariatric surgery was shown to improve or completely resolve steatosis, steatohepatitis, and fibrosis, the inclusion criteria for most of these studies were less rigorous (included observational studies) and may have potential bias (publication bias). Another Cochrane systematic review of 21 studies with histological outcomes of bariatric surgery looked at 21 prospective or retrospective cohort studies which showed improvement of steatosis or inflammation in most studies except for 4 studies that showed worsening of fibrosis [68]. Both of these systematic reviews showed improvement in liver function tests and certain risk factors for metabolic syndrome as well. Nevertheless, the fact that some studies showed worsening of liver fibrosis cannot be overlooked. Further long-term and well-designed prospective studies are needed to address these issues.

## 4. Weight Loss Medication

Orlistat and sibutramine have been studied as a treatment option for nonalcoholic fatty liver disease, though without much convincing results. Orlistat is a pancreatic lipase inhibitor that reduces absorption of free fatty acids [87]. Sibutramine is a centrally acting serotonin-norepinephrine reuptake inhibitor, which enhances satiety [88]. In a randomized prospective trial, 50 overweight patients with biopsy proven NASH were assigned to either 1400 kcal/day diet plus vitamin E 800 IU alone or with combination of orlistat for 9 months [89]. Both groups showed reduction in steatosis, necroinflammation, ballooning, and NAS in addition to reduction of weight and improvement in aminotransferases. However, independent of the groups, weight loss of greater than 5% led to improved insulin sensitivity and steatosis, while those that lost 9% or more of body weight had greater histological improvement. There were no significant differences between the two groups with respect to metabolic and histological markers, signifying that weight loss is responsible for histological improvement rather than the medication. In another double-blinded randomized placebo-controlled trial assessing the efficacy of orlistat in 52 patients with NAFLD (40 confirmed by biopsy), individuals were randomized to orlistat versus placebo for 6 months [90]. Mean weight loss was 8% with orlistat versus 6% in placebo group (difference was not significant). ALT improved in both groups, with greater improvement in orlistat group. Statistically significant reversal of steatosis by ultrasound was seen in orlistat group. Repeat biopsy performed in subset of subjects showed that both groups had 50% improvement in steatosis without significant improvement in fibrosis and inflammation.

Conversely, sibutramine was associated with 11.4% incidence of serious nonfatal myocardial infarction and nonfatal stroke as compared to placebo in the SCOUT trial [91]. Therefore, in 2010, FDA withdrew sibutramine from the US market due to risk of serious cardiovascular events [92] and many studies with sibutramine were discontinued due to adverse effects [93].

## 5. Antidiabetic Medications

### 5.1. Thiazolidinediones

Thiazolidinediones (TZDs) are peroxisomal proliferator activated receptor- (PPAR-) gamma agonists that promote hepatic fatty acid oxidation which increases hepatic lipogenesis and insulin sensitivity [94]. Most studies have shown improved biochemical efficacy with TZDs in NAFLD patients and some of them resulted in histological improvements such as steatosis and inflammation [95]. Nevertheless, these effects of TZDs do not seem to be sustained as some studies have noticed recurrence of NASH after discontinuation of TZDs [96, 97]. Furthermore, the important adverse effects associated with TZDs such as weight gain, edema, osteoporosis, and heart failure cannot be overlooked [97–99].

Rosiglitazone and pioglitazone are the two TZDs that have been studied the most. The FLIRT trial enrolled 63 biopsy proven NASH patients who received either rosiglitazone or placebo for 1 year [100]. Rosiglitazone was shown to improve steatosis, transaminase levels, adiponectin and insulin sensitivity, despite evidence of significant weight gain compared to placebo. There were no significant changes in fibrosis or NAS. In an open label study of 137 biopsy proven NASH subjects randomized to either rosiglitazone alone or combination of rosiglitazone and metformin versus combination of rosiglitazone and losartan for 48 weeks, there was no significant difference among the groups even though there was overall histological improvement in steatosis, inflammation, ballooning degeneration, and fibrosis as well as reduction of aminotransferase levels [101].

Pioglitazone has been studied extensively and was found to have more promising results in terms of liver enzymes and histological changes such as steatosis and inflammation. A study by Belfort et al. enrolled 55 patients with glucose intolerance or diabetes and biopsy proven NASH and assigned them to either hypocaloric diet plus pioglitazone or hypocaloric diet plus placebo for 6 months [69]. Pioglitazone group had a significant improvement in ALT, insulin sensitivity as well as steatosis, ballooning, and inflammation. Fibrosis reduction for pioglitazone group compared to placebo did not reach significance probably due to the small number of patients or short duration of study. In another randomized double-blind placebo-controlled trial with pioglitazone involving 74 nondiabetic subjects with NASH, pioglitazone group had significant improvement in hepatocellular injury and fibrosis after 12 months of treatment [98]. The PIVENS trial is a large randomized multicenter, double-blinded, placebo-controlled study of 247 nondiabetic patients with NASH who were assigned to one of three groups: pioglitazone 30 mg daily, vitamin E 800 IU daily, or placebo [70]. The primary endpoint of the study was decrease in NAS by at least 2 points and improvement in ballooning by 1 point. Vitamin E group as compared to other groups had significantly higher percentage of subjects with improvement in NASH (*p* < 0.001). Although pioglitazone group failed to reach primary endpoint, both treatment groups showed significant improvement in serum aminotransferases, reduction in steatosis, and lobular inflammation, but not fibrosis score. The failure to reach primary endpoint was possibly due to more subjects in the pioglitazone group having absence of ballooning at baseline while improvement in ballooning was one of the primary outcomes. Although most studies show no effect on reduction of fibrosis, thiazolidinediones such as pioglitazone may have a role in delaying progression of fibrosis in patients with NASH [27, 102]. Therefore, overall current data suggest that thiazolidinediones may be effective in treating NASH and are associated with significant improvement in biomarkers and histological markers such as steatosis and inflammation and possibly delaying fibrosis, although effects may be reversed after discontinuation of the medication [97]. Nevertheless, caution should be taken with regard to the use of TZDs due to the known cardiovascular adverse effects.

### 5.2. Metformin

Metformin improves insulin resistance by reducing hepatic gluconeogenesis and fatty acid oxidation, increasing peripheral and hepatic insulin sensitivity, decreasing intestinal glucose absorption, and lowering serum lipid concentration [103]. Recent animal studies have also suggested a possible role of metformin in prevention of hepatocellular carcinoma [104, 105]. Many studies have been done assessing the effectiveness of metformin in NAFLD patients, but the results have been inconsistent. Although some of the studies showed improvement in metabolic markers, aminotransferases, and liver histology, most of the benefits may be due to metformin's known effect of causing improvement in insulin sensitivity, weight loss, and other markers of metabolic syndrome [106, 107]. A randomized controlled trial involving 110 nondiabetic NAFLD patients for 12 months compared three groups: metformin, vitamin E, and prescriptive diet. It showed that metformin treatment is better than a prescriptive diet or vitamin E in terms of improvement in aminotransferases [108]. Histological analysis also showed modest improvement in hepatic steatosis and inflammation. On the contrary, other randomized controlled trials have failed to show histological benefit with metformin. Clearly, most studies show that metformin offers no additional histological or biochemical benefit other than its independent effect on insulin sensitivity and weight loss and therefore metformin is not recommended as first line treatment for nonalcoholic fatty liver disease [21].

### 5.3. Other Antidiabetic Agents

Studies have been done with meglitinides and incretin mimetics (GLP-1 analogs) to assess their effect on fatty liver. Meglitinide stimulates pancreatic insulin release and pancreatic beta cell growth. The data on use of meglitinide as treatment for NAFLD/NASH is scarce. Although some of the pilot studies involving these agents have shown improvement in metabolic biomarkers and histology, these studies involved very small sample sizes [109–111]. In a phase 2 trial with liraglutide (GLP-1 analog), 52 overweight subjects with NASH were randomly assigned to liraglutide versus placebo group [71]. Of the patients in liraglutide group, 39% had resolution of NASH compared to 9% in the placebo group. Only 9% of patients in the liraglutide group had worsening of fibrosis compared to 26% in the placebo group, which indicates a possible role of liraglutide in treating NASH and possibly delaying progression of fibrosis.

## 6. Antilipidemic Drugs

### 6.1. Statins

Lipid lowering drugs such as statins, niacin, fibric acid derivatives, ezetimibe, and n-3 PUFA have been studied for patients with hyperlipidemia and NAFLD (see Table 1). Dyslipidemia is one of the cardiovascular risk factors and an important component of metabolic syndrome which is a risk factor for NAFLD [112]. Although many studies with statins and NAFLD have been done, most of them lacked histological evaluation. Statin's beneficial effect on NAFLD has been proposed to be likely due to its anti-inflammatory, antioxidant, and antifibrinogenic properties [113, 114]. In a recent cross-sectional study, data analysis of 2578 patients who underwent liver ultrasound showed that 35.3% of them had hepatic steatosis while 990 subjects were either past or current user of statins [115]. Patients with BMI >27.5 kg/m^2^ and statin use for >2 years were associated with three times lower prevalence of steatosis.

Studies on efficacy of simvastatin have been inconsistent as in one study there was an improvement in serum ALT [116], while in another study there was no improvement in aminotransferases or histology [117]. In a multicenter, randomized, double-blind, placebo-controlled trial, 326 subjects with hyperlipidemia and chronic liver disease (NAFLD = 209 and hepatitis C = 117) were randomly assigned to either 80 mg of pravastatin or placebo for 36 weeks [118]. Compared to placebo, pravastatin was found to lower the lipid panel without causing significant changes in ALT. Studies involving atorvastatin have also shown significant decrease in aminotransferases, lipid levels [119, 120], and steatosis [121], while also delaying progression of NAFL to NASH [122]. In a randomized controlled trial involving patients with ultrasonographic evidence of NAFLD and hyperlipidemia, 63 subjects were assigned to 20 mg a day of atorvastatin, 62 subjects assigned to 200 mg a day of fenofibrate, and 61 subjects with combination of fenofibrate and atorvastatin [123]. The atorvastatin alone group and the combination group had a higher degree of biochemical improvement and ultrasonographic regression of NAFLD compared to fenofibrate alone. Therefore, most studies show consistency in improvement in transaminases while some show possible role in reversing steatosis and delaying progression of NAFLD to NASH. Overall, literature and guidelines support the use of statins in patients with NAFLD and hyperlipidemia [21].

### 6.2. Fibrates

Fibrates activate PPAR alpha and lipoprotein lipase and suppress apolipoprotein [124]. The evidence of beneficial effect of fibrates as a therapy for NAFLD is not sufficient. In a study of 40 patients with biopsy proven NASH (*n* = 24) and NAFL (*n* = 16) who were given 200 mg a day of fenofibrate for 48 weeks, there was significant improvement in triglycerides, alkaline phosphatase, gamma-glutamyl transpeptidase, and grade of ballooning degeneration [125]. However, there was no improvement in insulin resistance, transaminases, hepatic steatosis, necroinflammation, or fibrosis. In another randomized controlled trial, 46 patients with NASH were assigned to either 600 mg a day of gemfibrozil (*n* = 23) or placebo (*n* = 23) [126]. The treatment group had significantly lower liver enzymes, but no significant histological changes. The results of studies with fenofibrate as a therapeutic option for NAFLD are inconsistent. In fact, one study even found fenofibrate to cause increase in mortality from coronary heart disease compared to placebo, although the difference was not significant [127]. Total mortality was also slightly higher in the fenofibrate group compared to the placebo group (7.3% versus 6.6%; *p* = 0.18).

### 6.3. Niacin

Niacin has been suggested to prevent hepatic steatosis in experimental mice models [128]. However, in a randomized controlled double-blind trial involving 27 obese patients with NAFLD who underwent treatment with fenofibrate versus niacin versus placebo, both treatment groups failed to show any decrease in intrahepatic triglycerides [129]. Furthermore, some data actually showed niacin to have adverse hepatotoxic effect, ranging from mild elevation in aminotransferases to fulminant hepatic failure [130–132].

### 6.4. Niemann-Pick C1-Like 1 (NPC1L1) Inhibitor

In multiple studies, ezetimibe (NPC1L1 inhibitor) has been shown to improve biochemical parameters and histological markers of NAFLD [116, 133, 134], but most studies either lacked the control group or involved a small sample size. A recent randomized controlled trial involving 32 biopsy proven NAFLD patients given either 10 mg/day of ezetimibe or placebo for 6 months showed that ezetimibe group had significant improvement in ballooning score and fibrosis stage compared to the control group but did not have significant change in inflammation, steatosis, and overall NAS [135]. Nevertheless, this study also showed that ezetimibe was associated with significant increase in hepatic long-chain fatty acids, oxidative stress, insulin resistance, and HbA1c, which are known to be associated with development and progression of NAFLD.

### 6.5. Polyunsaturated Fatty Acids (PUFA)

Omega-3 belongs to a broader group of fats called polyunsaturated fatty acids (PUFA) that have been studied extensively as a treatment option for NAFLD. Studies have shown that NAFLD is associated with decreased hepatic PUFA [40, 136]. In one study involving 144 patients with NAFLD and hyperlipidemia, subjects were randomized to receive American Heart Association (AHA) diet plus PUFA versus AHA diet and placebo for 24 weeks [72]. In the PUFA group, there was complete regression of steatosis in 19.7% of patients and overall reduction of steatosis in 53.03% of patients along with significantly greater improvement in ALT compared to diet alone. More recently, in a double-blind randomized placebo-controlled clinical trial involving diabetic patients, 37 subjects with biopsy proven NASH were randomized to PUFA or placebo for 48 weeks [137]. The primary outcome for this study was improvement of NAS ≥2 points. Liver biopsy was done before and after completion of study. Surprisingly, in the placebo group, there was significant improvement in hepatic steatosis and NAS but worsening of lobular inflammation, whereas in the PUFA group, there was worsening of insulin resistance and there were no significant histological changes. Therefore, PUFA may not be a good option for patients with NASH and diabetes. Another double-blind, randomized, placebo-controlled trial with similar primary endpoint involved 34 subjects with biopsy proven NASH who were randomized to n-3 fish oil 3000 mg/day versus placebo for 1 year [73]. At the end of study, 4 of the 17 subjects in the treatment group and 3 of the 17 subjects in the placebo group had reduction in NAS of ≥2 points with no difference between the groups after adjusting for weight loss and other variables. Further studies with larger sample size are required to arrive at a firm conclusion regarding the beneficial effect of n-3 PUFAs.

## 7. Antioxidants

### 7.1. Vitamin E

Vitamin E has been shown to improve aminotransferases as well as histological markers in subjects with NASH (see Table 1). Some studies have also shown complete resolution of steatohepatitis, but there are mixed results on its effect on liver fibrosis. As described above in the pioglitazone section, the PIVENS trial, a randomized, multicenter, double-blind, placebo-controlled trial [70] showed that vitamin E when compared to placebo led to significantly higher rate of improvement in necroinflammation (43% versus 19%), although there was no significant improvement in fibrosis. Therefore, vitamin E can be used as treatment for nondiabetic NASH, but caution should still be taken as some studies show that vitamin E is associated with overall increased cardiovascular mortality [21, 138, 139] as well as increased risk of prostate cancer [140].

The effect of vitamin E on NAFLD has also been evaluated in many combination therapy studies. In a prospective, double-blind, randomized, placebo-controlled trial, 45 patients with biopsy proven NASH were enrolled to receive either vitamin E 1000 IU plus vitamin C 1000 mg or placebo for 6 months [74]. At the end of treatment, the vitamin E + C group had statistically significant improvement in fibrosis but no improvement in necroinflammation and ALT. Similar results were seen in a small study evaluating combination of vitamin E and pioglitazone compared to vitamin E alone [75]. In another combination study involving vitamin E with ursodeoxycholic acid (UDCA), 48 patients with biopsy proven NASH were randomly assigned to either UDCA + vitamin E or UDCA or placebo for two years [76]. Serum aminotransferases and steatosis improved significantly in the combination group compared to UDCA alone and placebo. Therefore, overall vitamin E shows histological beneficial effect on nondiabetic NASH subjects but also may have some additional benefits when combined with other agents, such as antioxidants, anti-inflammatory, and insulin sensitizing agents. Nevertheless, further studies with longer duration of treatment are necessary to conclude the therapeutic beneficial effect of vitamin E and its various combinations with other agents.

### 7.2. Other Antioxidants

Changes in methionine/folate metabolism may contribute to the development of steatosis [141]. S-adenosyl methionine (SAM) and betaine are nutritional supplements that have anti-TNF alpha, cytoprotective, antiapoptotic, and antisteatogenic activity and can cause reversal of insulin resistance [142, 143]. N-acetyl cysteine (NAC) is a glutathione precursor which increases glutathione in hepatocytes and limits the reactive oxygen species that causes hepatocellular injury [144]. Most studies involving these antioxidants have either a short duration or a small sample size. Further larger studies with longer duration treatment and histological analysis are required to confirm a beneficial effect of these antioxidants on NAFL/NASH.

## 8. Anti-Inflammatory Agents

Pentoxifylline is a xanthine derivative that inhibits TNF-alpha, which is a proinflammatory cytokine that has been shown to activate reactive oxygen species by lipid peroxidation and promote necroinflammation, fibrogenesis, hepatic insulin resistance, and apoptosis [145]. In a randomized placebo-controlled trial, 49 patients with biopsy proven NASH were randomized to pentoxifylline versus placebo for 1 year [77]. All patients in the treatment group had either improved NAS or no change. Compared to placebo, pentoxifylline group had significant improvement in ALT, steatosis, inflammation, and fibrosis but no change in ballooning. A decrease in NAS of ≥2 was seen in 50% of patients in pentoxifylline group versus 15.4% in placebo group. Additionally, 25% of NASH patients in the pentoxifylline group had resolution of NASH at the end of treatment. On the contrary, another randomized controlled trial of 30 patients with biopsy proven NASH failed to show any significant difference in improvement in aminotransferases and histology compared to placebo, although there was improvement in both parameters compared to baseline [146]. A recent meta-analysis showed that pentoxifylline significantly reduced serum aminotransferase, BMI, fasting glucose, steatosis, lobular inflammation, and fibrosis, but larger well-designed, randomized, placebo-controlled trials are still needed to confirm these results [147].

## 9. Probiotics

Bacterial overgrowth in the bowel is present in 50% of patients with NASH and changes in the intestinal bacterial content may be related to the pathogenesis of NASH due to enhanced intestinal permeability, activation of inflammatory cytokines, and absorption of endotoxins [148, 149]. Therefore, probiotics have been suggested as a treatment option in NASH patients [150]. However, some studies have shown worsening of steatosis with the use of probiotics [151]. In a double-blind randomized controlled trial, 30 patients with NAFLD diagnosed by biopsy were randomized to either* Lactobacillus bulgaricus* and* Streptococcus thermophilus* or placebo [78]. The treatment group had significant improvement in liver enzymes. In another study, 66 patients were randomly assigned to either* Bifidobacterium longum* with fructooligosaccharides (Fos) plus lifestyle modification (diet and exercise) or lifestyle modification alone for 24 weeks [79]. At the end of study, compared to the lifestyle modification group, the combination treatment group caused significant reduction in TNF-alpha, CRP, AST, HOMA-IR, steatosis, and NAS. In a meta-analysis of 4 double-blind randomized trials involving 134 biopsy proven NAFL/NASH patients, the authors concluded that probiotics significantly decreased total cholesterol, aminotransferases, HOMA-IR, and TNF-alpha [152].

## 10. Cytoprotective and Antiapoptotic Agents

Ursodeoxycholic acid (UDCA) has a beneficial effect on hepatobiliary diseases by its cytoprotective, immunomodulatory, and antiapoptotic effects [153]. The efficacy results of UDCA on NAFLD/NASH have been mixed, with some trials showing improvement in ALT and other histological markers such as steatosis, inflammation, and fibrosis [76, 154, 155], while others show no significant difference when compared to placebo [156–158]. In a recent systematic review of 12 randomized clinical trials, UDCA monotherapy was shown to improve liver enzymes, steatosis, and fibrosis in few studies, but UDCA combined with other drugs showed greater improvements in both steatosis and inflammation [155]. Moreover, there might be variation in treatment outcome with low and high dose UDCA [154]. Further evaluation of dose-dependent outcome is necessary.

## 11. Other Therapeutic Agents

### 11.1. RAAS (Renin-Angiotensin-Aldosterone System)

Emerging evidence, mainly in animal studies, has shown that inhibiting RAAS pathway decreases hepatic stellate cell activity, which in turn prevents fibrosis [159] and that ACE-inhibitors and angiotensin receptor blockers may be useful in treating NAFL/NASH as they may lead to decreased fibrosis [160]. However, not many studies have been done in humans with NAFLD to assess the effectiveness of ACE inhibitors or angiotensin receptor blockers (ARB). In one study, 54 patients with hypertension and NASH were randomly assigned to either telmisartan or valsartan; both groups showed improvement in transaminases and insulin resistance, while telmisartan was also shown to improve NAS and fibrosis [161]. Furthermore, in a recent cross-sectional study of 290 hypertensive patients with biopsy proven NAFLD, liver histology was compared in patients with and without RAS blocker; there was significantly less ballooning and lower fibrosis stage in patients on RAAS blockers [162]. Despite the promising results, larger randomized controlled studies are required to draw a conclusion on their beneficial effect.

### 11.2. Coffee

Coffee is rich in sources of bioactive phytochemicals including methylxanthines (caffeine), amino acids, phenolic acids, and polyphenols which may protect against liver disease. A study involved 195 patients participating in a questionnaire about coffee and espresso as well as other caffeinated drinks and chocolate [80]. All patients underwent liver biopsies and compared to espresso, regular coffee consumption was associated with decreased odds ratio of liver fibrosis. Furthermore, espresso was associated with the presence of metabolic syndrome. Again, this study suggests possible beneficial effect of coffee and not caffeine, which is also present in espresso, but espresso failed to demonstrate the same beneficial effect. In a recent meta-analysis of 16 studies comprising 3153 cases of hepatocellular carcinoma, the risk of HCC was found to be reduced by 40% with coffee intake compared to no coffee [163]. Further well organized larger studies are required to draw any conclusion.

### 11.3. Vitamin D

There is not enough data assessing the effect of vitamin D supplementation on NAFLD, even though studies have shown vitamin D deficiency is associated with development of NAFLD [164, 165]. A study of 60 patients with biopsy proven NAFLD and 60 healthy controls shows that, compared to controls, NAFLD patients had a significant decrease in vitamin D levels and that levels of vitamin D also negatively correlated with histological severity of steatosis, necroinflammation, and fibrosis independent of other variables or presence of metabolic syndrome [166]. Despite multiple studies suggesting association of NAFLD with low vitamin D, supplementation has not been shown to be very promising (see Table 1). In a recent randomized, double-blind, placebo-controlled trial, 53 patients with NAFLD were given 50,000 IU every two weeks or placebo for a total study duration of 4 months and the results showed no significant difference in insulin resistance, TNF-alpha, or aminotransferases [81].

### 11.4. Phlebotomy

Although there has been a significant recent interest in the role of iron in NAFLD, the results have been conflicting. A recent study showed that patients who were suspected to have NAFLD had higher body iron and a greater hemoglobin level [167]. Another study involving data collected from 1,201 biopsy proven adult NAFLD patients showed that although ferritin level was significantly associated with steatosis and fibrosis, it was not shown to be a good predictor of fibrosis [168]. Similar results were seen in a multicenter study involving 1014 patients with liver biopsy-confirmed NAFLD [169]. In a phase II clinical trial involving 31 patients with biopsy proven NAFLD, all subjects underwent phlebotomy to achieve ferritin level ≤50 *μ*g/liter [170]. Repeat biopsy was done 6 months after treatment. Iron reduction correlated with significant reduction in overall NAS, although there was no significant change in the individual histological features of steatosis, inflammation, ballooning, or fibrosis. In summary, although serum ferritin seems to lack diagnostic accuracy for fibrosis, phlebotomy as a therapeutic option needs to be further studied.

### 11.5. Herbal Medications

Recently, herbal therapy has been studied as treatment option for NAFLD as some animal models have shown a beneficial effect of correcting markers of inflammation, metabolic risk factors, transaminases, and histological markers such as steatosis. Herbal supplements should still be cautiously used and further investigated for adverse effects prior to considering for NAFLD treatment.

Milk thistles, also known as* Silybum marianum* or silymarin, have been shown to have antioxidant, anti-inflammatory, and antifibrogenic activity along with its ability to help with liver cell regeneration [171–173]. In a randomized double-blind, controlled trial, silybin (major active constituent of silymarin) combined with vitamin E and phospholipids was shown to significantly improve steatosis [82]. Furthermore, in another multicenter, double-blind clinical trial, 138 patients were randomized to either silybin plus phosphatidylcholine or placebo for 12 months [83]. Patients receiving the combination treatment showed a significant improvement in liver enzymes, HOMA-IR, and liver histology. Therefore, silybin as a combination treatment seems to have potential therapeutic ability, but further studies are required to assess its beneficial effect on NAFLD/NASH.

## 12. Upcoming Drugs

### 12.1. Bile Acid Receptors

Farnesoid X receptor (FXR) is a bile acid nuclear receptor that is highly expressed in the liver, intestine, kidney, and adipose tissue (see Table 2). These receptors have been proposed to have a role in development of hepatic steatosis. Stimulation of these receptors improves insulin sensitivity and decreases hepatic steatosis, inflammation, and fibrosis [174–176]. Obeticholic acid (OCA) is an FXR agonist which has been tested as a treatment option for NAFLD. In a double-blind, placebo-controlled trial, treatment with OCA for 6 weeks led to improvement in insulin sensitivity and reduction in markers of liver inflammation and fibrosis [84]. In FLINT trial, which is a multicenter, double-blind, placebo-controlled trial, 283 noncirrhotic NASH patients were randomly assigned to OCA versus placebo [85]. In this study, OCA compared to placebo group had significant improvement in aminotransferases, HOMA-IR, and all markers of NAS (*p* = 0.002). Other than worsening of lipid panel which might require addition of a statin, this study had mostly positive outcomes. In fact, the trial was stopped early because of efficacy based on the high statistically significant improvement in liver histology. A phase 3 international trial, also known as REGENERATE trial, assessing the effect of OCA on patients with NASH with fibrosis is underway [177].

### 12.2. PPAR Alpha/Delta Agonist

Elafibranor (GFT505) is a PPAR alpha/delta agonist which has anti-inflammatory and antifibrotic properties [178] and has been shown to improve liver function and insulin sensitivity [179, 180]. In a phase 2 multicenter randomized controlled trial, 274 subjects with histologically proven NASH received either GFT505 80 mg/daily or 120 mg/daily or placebo for one year [clinicaltrials.gov NCT01694849]. Primary outcome of the study was resolution of NASH without worsening of fibrosis. Compared to placebo group, relative risk of achieving primary endpoint in the 120 mg and 80 mg arms was 1.94 (*p* = 0.027) and 1.68 (*p* = 0.091), respectively. In the 120 mg group, there were significant improvements in all histological markers including fibrosis. Additionally, compared to placebo, GFT505 leads to significant improvement in metabolic markers. This study showed that GFT505 produces a dose-dependent improvement in histology of patients with NASH. Currently, phase 3 trial with GFT505 is underway.

### 12.3. Other Drugs

Among the other upcoming drugs which are being studied in NASH patients is cysteamine bitartrate (see Table 2). In an open label phase 2a clinical trial involving patients with biopsy proven NAFLD with elevated aminotransferases, participants received treatment with cysteamine bitartrate for six months and result showed marked decline in ALT and AST levels in all patients as well as significant decrease in cytokeratin and increase in adiponectin levels [181]. In another phase 2 placebo-controlled trial with caspase inhibitor involving 124 patients with biopsy proven NASH, result showed improvement in ALT and cytokeratin [182]. Another new drug with great potentials includes simtuzumab, which is a monoclonal antibody against lysyl oxidase-like 2 (LOXL2) with antineoplastic activity now being investigated in clinical trials as treatment for NASH and advanced fibrosis [clinicaltrials.gov NCT01672879].

## 13. Conclusion

This review examined the most recent data available for treatment of NAFLD. Along with reviewing the available data on pharmaceutical approach, we also reviewed the herbal, dietary, and nutritional aspects of management of fatty liver. Weight loss with diet and exercise seems to be the mainstay of treatment. Among the nonpharmacological approaches, regular exercise along with healthy diet, possibly incorporation of mono- or polyunsaturated fatty acids, and/or Mediterranean diet seems to be beneficial even without significant weight loss, although weight loss will definitely improve metabolic risk factors. If compliance is a hindrance for achieving lifestyle modification, incorporation of counseling and support group will help facilitate and achieve positive outcome. Amongst pharmacological agents, evidence suggests that vitamin E and pioglitazone are the most effective drugs in treating NASH, but also at the expense of potential adverse effects. Vitamin E may have even better efficacy when combined with other agents. Ursodeoxycholic acid (UDCA), pentoxifylline, and herbal medications such as milk thistle may also have potential therapeutic benefit, although results are inconclusive. Interestingly, coffee consumption with or without caffeine seems to have beneficial effect on nonalcoholic fatty liver disease, though most studies involved retrospective questionnaires. Furthermore, recent advances in phase 2 and 3 trials have definitely shown promising results, with few agents having the potential of becoming future therapeutic agent for NASH.

## Figures and Tables

**Table 1:** Summary of treatment options for NAFL/NASH.

Treatment strategy	Medication/intervention	Comment
Lifestyle modification	Weight loss	5–10% weight loss can lead to biochemical and histological improvement [24, 25]
	Hypocaloric diet alone	Better response when combined with exercise and reduced carbohydrate and fat [54, 55]
	Dietary composition	Diet should include addition of PUFA and decreased saturated and transfatty acids [40–42]. Mediterranean diet has shown improvement in ALT, insulin levels, and histological markers [59, 60]
	Exercise alone	Effective even without weight loss [38, 61]
	Diet plus exercise	Greatest beneficial effect [24, 64, 65]
	Bariatric surgery	Could be beneficial in reversing NASH [22, 66]; some studies show worsening fibrosis [67, 68]
	Orlistat	Not enough evidence to support or oppose its use
	Sibutramine	FDA withdrew sibutramine from market due to cardiovascular events

Insulin sensitivity	Thiazolidinediones	For patients with NASH, they improve insulin resistance, biomarkers, steatosis, and inflammation [69, 70]; caution should be taken in regard to cardiovascular risks
	Metformin	It offers no additional biochemical or histological benefits
	Meglitinides and incretin mimetics	GLP-1 analog has shown histological improvement in phase 2 trial [71]

Antilipidemic	Statins	Decrease in transaminases, possible role in delaying progression from NAFL to NASH; safe to use in patients with NAFL and NASH
	Fenofibrate	Possible increased mortality risk in NAFLD patients
	Niacin	May be hepatotoxic
	NPC1L1 inhibitor	Not enough evidence to support or oppose its use
	n-3 polyunsaturated fatty acids	May help reverse NAFLD according to some studies [72, 73]

Antioxidants	Vitamin E	Improves histological markers in nondiabetic NASH [70]; may have greater benefit when combined with other agents [74–76]; may be associated with increased risk for cardiovascular events and prostate cancer
	SAM, NAC, and betaine	Not enough evidence to support or oppose its use

Anti-inflammatory	Pentoxifylline	May improve liver enzymes and histology [77]

Probiotics		May improve liver enzymes, insulin resistance, and anti-inflammatory markers [78, 79]

Cytoprotective and antiapoptotic agents	Ursodeoxycholic acid	May improve liver enzymes and histology, but further well-designed studies are needed

Other	RAAS	Small studies show improvement in liver enzymes and histology, but larger randomized controlled studies are warranted
	Coffee	Coffee is associated with decreased prevalence of NAFLD and may have a role in delaying progression of fibrosis [80]
	Vitamin D	Replacement does not show improvement [81]
	Phlebotomy	Not enough evidence to support or oppose its therapeutic use

Herbal	Milk thistle	May improve liver chemistry and histology especially in combination with other agents such as vitamin E and phospholipids [82, 83]

NASH: nonalcoholic steatohepatitis; PUFA: polyunsaturated fatty acids; FDA: Food and Drug Administration; NAFL: nonalcoholic fatty liver; NAFLD: nonalcoholic fatty liver disease; NPC1L1: Niemann-Pick C1-Like 1; SAM: S-adenosyl methionine; NAC: N-acetyl cysteine; RAAS: Renin-Angiotensin-Aldosterone System.

**Table 2:** Summary of upcoming therapeutic agents for NAFL/NASH.

Treatment strategy	Medication	Comment
Bile Acid Receptors (FXR)	OCA	Has been shown to improve aminotransferases, insulin resistance, and NAS but can worsen lipid panel [84, 85]

PPAR alpha/delta agonist	Elafibranor	Can improve metabolic risk factors and lead to resolution of NASH [https://clinicaltrials.gov NCT01694849]

Other agents	Cysteamine bitartrate, caspase inhibitor, and simtuzumab	Had promising results in phase 2 trials and some are still undergoing clinical trials

FXR: farnesoid receptor; OCA: obeticholic acid.
